# 2-{[(4-{[(2-Hy­droxy­phen­yl)(phen­yl)methyl­idene]amino}­but­yl)imino](phen­yl)meth­yl}phenol

**DOI:** 10.1107/S1600536811055905

**Published:** 2012-01-07

**Authors:** Arezoo Jamshidvand, Reza Kia, Hadi Kargar, Muhammad Nawaz Tahir

**Affiliations:** a Department of Chemistry, Payame Noor University, PO BOX 19395-3697 Tehran, I. R. of Iran; b X-ray Crystallography Lab., Plasma Physics Research Center, Science and Research Branch, Islamic Azad University, Tehran, Iran; c Department of Chemistry, Science and Research Branch, Islamic Azad University, Tehran, Iran; d Department of Physics, University of Sargodha, Punjab, Pakistan

## Abstract

The asymmetric unit of the title compound, C_30_H_28_N_2_O_2_, comprises half of a potential tetra­dentate Schiff base ligand; an inversion centre is situtated at the center of the butane­diamine spacer. The central methyl­ene segment of the diamine spacer is disordered over two positions with a refined site-occupancy ratio of 0.651 (7):0.349 (7). The phenyl ring and the hy­droxy-substituted benzene ring are almost perpendicular to each other, with a dihedral angle of 87.90 (8) Å. Intra­molecular O—H⋯N hydrogen bonds make *S*(6) ring motifs.

## Related literature

For standard bond lengths, see: Allen *et al.* (1987 ▶). For hydrogen bond motifs, see: Bernstein *et al.* (1995 ▶). For background to Schiff bases in coordination chemistry, see: Granovski *et al.* (1993 ▶); Kargar *et al.* (2009 ▶). For a related structure, see: Friscic *et al.* (1998 ▶).
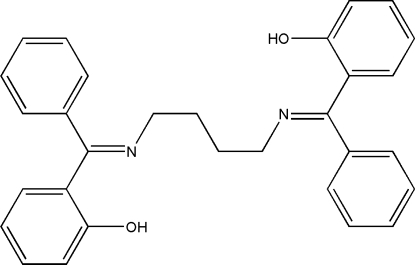


## Experimental

### 

#### Crystal data

C_30_H_28_N_2_O_2_
*M* *_r_* = 448.54
Monoclinic, 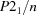

*a* = 11.5720 (3) Å
*b* = 7.7803 (2) Å
*c* = 13.3914 (4) Å
β = 95.774 (2)°
*V* = 1199.56 (6) Å^3^
*Z* = 2
Mo *K*α radiation
μ = 0.08 mm^−1^
*T* = 291 K
0.25 × 0.16 × 0.12 mm

#### Data collection

Bruker SMART APEXII CCD area-detector diffractometer
Absorption correction: multi-scan (*SADABS*; Bruker, 2005 ▶) *T*_min_ = 0.250, *T*_max_ = 0.459
10739 measured reflections
2951 independent reflections
1706 reflections with *I* > 2σ(*I*)
*R*_int_ = 0.027

#### Refinement

*R*[*F* ^2^ > 2σ(*F* ^2^)] = 0.045
*wR*(*F* ^2^) = 0.127
*S* = 1.02
2951 reflections
165 parameters
H-atom parameters constrained
Δρ_max_ = 0.16 e Å^−3^
Δρ_min_ = −0.13 e Å^−3^

### 

Data collection: *APEX2* (Bruker, 2005 ▶); cell refinement: *SAINT* (Bruker, 2005 ▶); data reduction: *SAINT*; program(s) used to solve structure: *SHELXS97* (Sheldrick, 2008 ▶); program(s) used to refine structure: *SHELXL97* (Sheldrick, 2008 ▶); molecular graphics: *SHELXTL* (Sheldrick, 2008 ▶); software used to prepare material for publication: *SHELXTL* and *PLATON* (Spek, 2009 ▶).

## Supplementary Material

Crystal structure: contains datablock(s) global, I. DOI: 10.1107/S1600536811055905/su2355sup1.cif

Structure factors: contains datablock(s) I. DOI: 10.1107/S1600536811055905/su2355Isup2.hkl

Supplementary material file. DOI: 10.1107/S1600536811055905/su2355Isup3.cml

Additional supplementary materials:  crystallographic information; 3D view; checkCIF report

## Figures and Tables

**Table 1:** Hydrogen-bond geometry (Å, °)

*D*—H⋯*A*	*D*—H	H⋯*A*	*D*⋯*A*	*D*—H⋯*A*
O1—H1⋯N1	0.82	1.80	2.5328 (16)	148

**Fig. 1.**
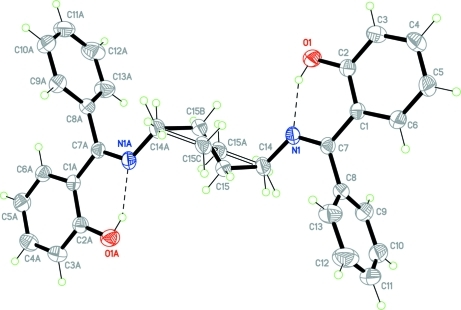
A view of the molecular structure of the title compound, showing 40% probability displacement ellipsoids and the atomic numbering. The dashed lines show the intramolecular O-H···N hydrogen bonds [symmetry code: (A) -x, -y, -z].

## supplementary crystallographic information

### Comment

Schiff base ligands are one of the most prevalent systems in coordination chemistry (Granovski *et al.*, 1993; Kargar *et al.*, 2009). As part of a general study of potential tetradenate Schiff bases (Kargar *et al.*, 2009), we have synthesized the title compound and report herein on its crystal structure.

The asymmetric unit of the title compound, Fig. 1, comprises half of a potential tetradentate Schiff base ligand. The inversion centre is situtated at the center of the butanediamine spacer. The bond lengths (Allen *et al.*, 1987) and angles are within the normal ranges and are comparable to those reported for a related structure (Friscic *et al.*, 1998).

There are intramolecular O—H···N hydrogen bonds (Table 1) making S(6) ring motifs (Bernstein *et al.*, 1995). The phenyl ring and the hydroxy-substituted benzene ring are almost perpendicular to each other with a dihedral angle of 87.90 (8)Å. The central methylene segment (C15) of the diamine spacer was disordered over two positions with a refined site occupancy ratio of 0.651 (7)/0.349 (7).

### Experimental

The title compound was synthesized by adding 2-hydroxybenzophenone (2 mmol) to a solution of 1,4-butylenediamine (1 mmol) in ethanol (30 ml). The mixture was refluxed with stirring for 30 min. The resultant solution was filtered. Yellow single crystals of the title compound, suitable for *X*-ray structure determination, were recrystallized from ethanol by slow evaporation of the solvents at room temperature over several days. The sample was hygroscopic and for the data collection it was sealed in fine glass cappilary under an inert atmosphere.

### Refinement

The OH and C-bound H-atoms were included in calculated positions and treated as riding atoms: O-H = 0.82 Å, C-H = 0.93 and 0.97 Å for CH and CH_2_ H-atoms, respectively, with U_iso_(H) = k × U_eq_(C), where k = 1.5 for OH, and k = 1.2 for all other H-atoms. The methylene segment (C15) of the diamine spacer was disordered over two positions with a refined site occupancy ratio 0.651 (7)/0.349 (7).

### Crystal data

**Table:**

C_30_H_28_N_2_O_2_	*F*(000) = 476
*M**_r_* = 448.54	*D*_x_ = 1.242 Mg m^−^^3^
Monoclinic, *P*2_1_/*n*	Mo *K*α radiation, λ = 0.71073 Å
Hall symbol: -P 2yn	Cell parameters from 2370 reflections
*a* = 11.5720 (3) Å	θ = 2.5–27.5°
*b* = 7.7803 (2) Å	µ = 0.08 mm^−^^1^
*c* = 13.3914 (4) Å	*T* = 291 K
β = 95.774 (2)°	Block, yellow
*V* = 1199.56 (6) Å^3^	0.25 × 0.16 × 0.12 mm
*Z* = 2	

### Data collection

**Table:**

Bruker SMART APEXII CCD area-detector diffractometer	2951 independent reflections
Radiation source: fine-focus sealed tube	1706 reflections with *I* > 2σ(*I*)
graphite	*R*_int_ = 0.027
φ and ω scans	θ_max_ = 28.3°, θ_min_ = 2.2°
Absorption correction: multi-scan (*SADABS*; Bruker, 2005)	*h* = −15→11
*T*_min_ = 0.250, *T*_max_ = 0.459	*k* = −10→9
10739 measured reflections	*l* = −17→17

### Refinement

**Table:**

Refinement on *F*^2^	Primary atom site location: structure-invariant direct methods
Least-squares matrix: full	Secondary atom site location: difference Fourier map
*R*[*F*^2^ > 2σ(*F*^2^)] = 0.045	Hydrogen site location: inferred from neighbouring sites
*wR*(*F*^2^) = 0.127	H-atom parameters constrained
*S* = 1.02	*w* = 1/[σ^2^(*F*_o_^2^) + (0.0565*P*)^2^ + 0.0873*P*] where *P* = (*F*_o_^2^ + 2*F*_c_^2^)/3
2951 reflections	(Δ/σ)_max_ < 0.001
165 parameters	Δρ_max_ = 0.16 e Å^−^^3^
0 restraints	Δρ_min_ = −0.13 e Å^−^^3^

### Special details

**Table:**

Geometry. All esds (except the esd in the dihedral angle between two l.s. planes) are estimated using the full covariance matrix. The cell esds are taken into account individually in the estimation of esds in distances, angles and torsion angles; correlations between esds in cell parameters are only used when they are defined by crystal symmetry. An approximate (isotropic) treatment of cell esds is used for estimating esds involving l.s. planes.
Refinement. Refinement of F^2^ against ALL reflections. The weighted R-factor wR and goodness of fit S are based on F^2^, conventional R-factors R are based on F, with F set to zero for negative F^2^. The threshold expression of F^2^ > 2sigma(F^2^) is used only for calculating R-factors(gt) etc. and is not relevant to the choice of reflections for refinement. R-factors based on F^2^ are statistically about twice as large as those based on F, and R- factors based on ALL data will be even larger.

### Fractional atomic coordinates and isotropic or equivalent isotropic displacement parameters (Å^2^)

**Table:**

	*x*	*y*	*z*	*U*_iso_*/*U*_eq_	Occ. (<1)
O1	0.08671 (10)	−0.51502 (14)	0.09514 (8)	0.0613 (3)	
H1	0.0426	−0.4348	0.0797	0.092*	
N1	−0.04017 (10)	−0.25674 (14)	0.12091 (9)	0.0500 (3)	
C1	0.04494 (11)	−0.42065 (16)	0.25836 (10)	0.0428 (3)	
C2	0.10105 (12)	−0.53074 (17)	0.19507 (11)	0.0469 (4)	
C3	0.17362 (13)	−0.6602 (2)	0.23703 (13)	0.0595 (4)	
H3	0.2106	−0.7332	0.1954	0.071*	
C4	0.19106 (14)	−0.6812 (2)	0.33870 (13)	0.0654 (5)	
H4	0.2400	−0.7681	0.3655	0.078*	
C5	0.13674 (14)	−0.5747 (2)	0.40225 (12)	0.0631 (4)	
H5	0.1488	−0.5897	0.4714	0.076*	
C6	0.06477 (13)	−0.44660 (19)	0.36208 (11)	0.0535 (4)	
H6	0.0283	−0.3752	0.4049	0.064*	
C7	−0.03030 (11)	−0.28060 (16)	0.21646 (10)	0.0436 (3)	
C8	−0.09188 (12)	−0.17392 (18)	0.28775 (10)	0.0481 (4)	
C9	−0.19981 (14)	−0.2220 (2)	0.31276 (13)	0.0633 (4)	
H9	−0.2363	−0.3183	0.2830	0.076*	
C10	−0.25395 (17)	−0.1277 (3)	0.38186 (15)	0.0814 (6)	
H10	−0.3269	−0.1610	0.3982	0.098*	
C11	−0.2023 (2)	0.0122 (3)	0.42610 (15)	0.0852 (6)	
H11	−0.2384	0.0731	0.4741	0.102*	
C12	−0.0963 (2)	0.0639 (3)	0.39977 (16)	0.0891 (6)	
H12	−0.0615	0.1621	0.4288	0.107*	
C13	−0.04048 (15)	−0.0282 (2)	0.33051 (13)	0.0701 (5)	
H13	0.0313	0.0080	0.3129	0.084*	
C14	−0.11235 (13)	−0.1200 (2)	0.07252 (12)	0.0592 (4)	
H14A	−0.1640	−0.0764	0.1194	0.071*	0.651 (7)
H14B	−0.1598	−0.1670	0.0152	0.071*	0.651 (7)
H14C	−0.1226	−0.0301	0.1200	0.071*	0.349 (7)
H14D	−0.1874	−0.1654	0.0494	0.071*	0.349 (7)
C15	−0.0405 (3)	0.0257 (4)	0.0383 (3)	0.0529 (10)	0.651 (7)
H15A	0.0050	0.0741	0.0963	0.063*	0.651 (7)
H15B	−0.0923	0.1148	0.0096	0.063*	0.651 (7)
C15A	−0.0554 (5)	−0.0468 (8)	−0.0156 (5)	0.0503 (19)	0.349 (7)
H15C	−0.0394	−0.1399	−0.0604	0.060*	0.349 (7)
H15D	−0.1092	0.0314	−0.0525	0.060*	0.349 (7)

### Atomic displacement parameters (Å^2^)

**Table:**

	*U*^11^	*U*^22^	*U*^33^	*U*^12^	*U*^13^	*U*^23^
O1	0.0729 (8)	0.0651 (7)	0.0466 (7)	0.0139 (5)	0.0090 (5)	−0.0025 (5)
N1	0.0544 (8)	0.0472 (7)	0.0489 (8)	0.0007 (5)	0.0084 (6)	0.0125 (5)
C1	0.0432 (8)	0.0421 (7)	0.0436 (8)	−0.0038 (6)	0.0061 (6)	0.0031 (6)
C2	0.0466 (8)	0.0459 (8)	0.0481 (9)	−0.0032 (6)	0.0045 (6)	−0.0001 (6)
C3	0.0580 (10)	0.0555 (9)	0.0646 (11)	0.0104 (7)	0.0036 (8)	−0.0051 (8)
C4	0.0620 (11)	0.0620 (10)	0.0700 (12)	0.0148 (8)	−0.0038 (8)	0.0092 (9)
C5	0.0661 (11)	0.0728 (11)	0.0488 (9)	0.0081 (8)	−0.0019 (8)	0.0126 (8)
C6	0.0565 (10)	0.0591 (9)	0.0455 (9)	0.0037 (7)	0.0084 (7)	0.0026 (7)
C7	0.0418 (8)	0.0423 (8)	0.0473 (9)	−0.0051 (6)	0.0080 (6)	0.0048 (6)
C8	0.0497 (9)	0.0467 (8)	0.0483 (8)	0.0060 (6)	0.0074 (7)	0.0050 (6)
C9	0.0580 (10)	0.0638 (10)	0.0709 (11)	0.0014 (8)	0.0199 (8)	0.0080 (8)
C10	0.0747 (13)	0.0904 (15)	0.0849 (14)	0.0239 (11)	0.0370 (11)	0.0235 (12)
C11	0.1070 (17)	0.0843 (15)	0.0679 (13)	0.0450 (13)	0.0262 (12)	0.0066 (11)
C12	0.1096 (17)	0.0709 (12)	0.0858 (15)	0.0154 (11)	0.0050 (12)	−0.0255 (10)
C13	0.0653 (11)	0.0635 (10)	0.0822 (13)	−0.0011 (8)	0.0106 (9)	−0.0142 (9)
C14	0.0587 (10)	0.0596 (10)	0.0599 (10)	0.0060 (7)	0.0080 (7)	0.0204 (8)
C15	0.063 (2)	0.0466 (16)	0.0497 (19)	0.0073 (13)	0.0104 (14)	0.0057 (15)
C15A	0.057 (4)	0.048 (3)	0.044 (4)	0.008 (2)	−0.003 (2)	0.009 (3)

### Geometric parameters (Å, °)

**Table:**

O1—C2	1.3373 (17)	C10—C11	1.350 (3)
O1—H1	0.8200	C10—H10	0.9300
N1—C7	1.2867 (17)	C11—C12	1.371 (3)
N1—C14	1.4631 (18)	C11—H11	0.9300
C1—C6	1.3994 (19)	C12—C13	1.382 (3)
C1—C2	1.4087 (19)	C12—H12	0.9300
C1—C7	1.4703 (19)	C13—H13	0.9300
C2—C3	1.393 (2)	C14—C15	1.504 (3)
C3—C4	1.366 (2)	C14—C15A	1.519 (6)
C3—H3	0.9300	C14—H14A	0.9700
C4—C5	1.383 (2)	C14—H14B	0.9700
C4—H4	0.9300	C14—H14C	0.9599
C5—C6	1.374 (2)	C14—H14D	0.9600
C5—H5	0.9300	C15—C15^i^	1.512 (7)
C6—H6	0.9300	C15—H15A	0.9700
C7—C8	1.4984 (19)	C15—H15B	0.9700
C8—C9	1.377 (2)	C15A—C15A^i^	1.497 (13)
C8—C13	1.378 (2)	C15A—H15C	0.9700
C9—C10	1.380 (2)	C15A—H15D	0.9700
C9—H9	0.9300		
			
C2—O1—H1	109.5	C8—C13—H13	120.2
C7—N1—C14	122.34 (13)	C12—C13—H13	120.2
C6—C1—C2	118.03 (12)	N1—C14—C15	112.01 (15)
C6—C1—C7	121.16 (13)	N1—C14—C15A	110.1 (2)
C2—C1—C7	120.80 (12)	C15—C14—C15A	35.4 (2)
O1—C2—C3	118.61 (13)	N1—C14—H14A	109.2
O1—C2—C1	121.88 (12)	C15—C14—H14A	109.2
C3—C2—C1	119.51 (13)	C15A—C14—H14A	135.7
C4—C3—C2	120.74 (14)	N1—C14—H14B	109.2
C4—C3—H3	119.6	C15—C14—H14B	109.2
C2—C3—H3	119.6	C15A—C14—H14B	77.4
C3—C4—C5	120.72 (14)	H14A—C14—H14B	107.9
C3—C4—H4	119.6	N1—C14—H14C	109.9
C5—C4—H4	119.6	C15—C14—H14C	76.2
C6—C5—C4	119.27 (15)	C15A—C14—H14C	109.4
C6—C5—H5	120.4	H14A—C14—H14C	36.2
C4—C5—H5	120.4	H14B—C14—H14C	134.5
C5—C6—C1	121.72 (14)	N1—C14—H14D	109.4
C5—C6—H6	119.1	C15—C14—H14D	133.6
C1—C6—H6	119.1	C15A—C14—H14D	109.7
N1—C7—C1	118.45 (12)	H14A—C14—H14D	75.0
N1—C7—C8	123.60 (12)	H14B—C14—H14D	35.3
C1—C7—C8	117.95 (12)	H14C—C14—H14D	108.3
C9—C8—C13	119.12 (15)	C14—C15—C15^i^	114.0 (4)
C9—C8—C7	120.37 (14)	C14—C15—H14C	36.2
C13—C8—C7	120.50 (13)	C15^i^—C15—H14C	148.7
C8—C9—C10	120.18 (17)	C14—C15—H15A	108.7
C8—C9—H9	119.9	C15^i^—C15—H15A	108.7
C10—C9—H9	119.9	H14C—C15—H15A	82.4
C11—C10—C9	120.84 (19)	C14—C15—H15B	108.7
C11—C10—H10	119.6	C15^i^—C15—H15B	108.7
C9—C10—H10	119.6	H14C—C15—H15B	94.6
C10—C11—C12	119.46 (18)	H15A—C15—H15B	107.6
C10—C11—H11	120.3	C15A^i^—C15A—C14	113.1 (7)
C12—C11—H11	120.3	C15A^i^—C15A—H15C	109.0
C11—C12—C13	120.74 (19)	C14—C15A—H15C	109.0
C11—C12—H12	119.6	C15A^i^—C15A—H15D	109.0
C13—C12—H12	119.6	C14—C15A—H15D	109.0
C8—C13—C12	119.62 (18)	H15C—C15A—H15D	107.8

Symmetry codes: (i) −*x*, −*y*, −*z*.

### Hydrogen-bond geometry (Å, °)

**Table:**

*D*—H···*A*	*D*—H	H···*A*	*D*···*A*	*D*—H···*A*
O1—H1···N1	0.82	1.80	2.5328 (16)	148
